# Complexity at mesoscopic lengthscale

**DOI:** 10.1107/S2052252515014670

**Published:** 2015-08-20

**Authors:** T. Egami

**Affiliations:** a Joint Institute for Neutron Sciences, Department of Materials Science and Engineering and Department of Physics and Astronomy, University of Tennessee, Knoxville, TN 37996, USA; b Oak Ridge National Laboratory, Oak Ridge, TN 37831, USA

**Keywords:** doped ceria, disorder, pair distribution function, high-resolution X-ray powder diffraction, percolation, hierarchy, solid electrolytes, electron spin resonance

## Abstract

Modern materials are often complex in the structure at mesoscale. The method of pair-density function (PDF) is a powerful tool to characterize mesoscopic structure, bridging short- and long-range structures.

At the dawn of crystallography the crystals studied by diffraction were simple, with just a few atoms in the unit cell. Since then the number of atoms in the unit cells of studied crystals has grown to many thousands in molecular and biological crystals, but the crystals are still single crystals. On the other hand liquids and glasses have been studied as well, but they are usually assumed to be uniform on a mesoscopic lengthscale. However, today we deal more frequently with matter with mesoscopic complexity, because advanced materials often perform better because of such complexity. Engineering at the nanoscale has endowed us with the freedom to design new and complex materials. Characterization tools also evolved with this progress. Crystallography was created for the study of perfect crystals, whereas deviations from perfect periodicity can be seen by diffuse scattering. To detect mesoscopic foreign objects, such as inclusions, small-angle-scattering (SAS) methods were developed. Furthermore, to study local structure within nearest neighbors, X-ray absorption spectroscopy (EXAFS and XANES), NMR and other techniques were added to the toolbox.

But these techniques have many blind spots. For instance SAS is sensitive only to density contrast, and density-preserving changes, such as shear transformations, cannot be seen by SAS. Diffuse scattering tells us the lengthscale of deviations from periodicity, but unless it is studied over many Brillouin zones it is difficult to find out what kind of deviations are actually taking place. Among these tools the pair-density function (PDF) method is probably the most versatile in capturing the essence of mesoscopic structure. The PDF is obtained by Fourier-transforming the total structure function, *S*(*Q*). Because both Bragg and diffuse intensities are included, it can describe periodic as well as aperiodic structure. It is of course not a perfect solution. For instance, because of spherical averaging the orientational information is lost. However, at least all the diffuse intensities are captured, so that it is more accurate in that sense.

The origin of the PDF method is as old as crystallography (Egami & Billinge, 2012 ▸). The concept of the PDF, a two-body positional correlation function, was used by Zernike and Prins in 1927 to explain diffraction patterns, for instance those from one-dimensional molecules (Zernike & Prins, 1927 ▸). Warren was the pioneer of using this approach to determine the structure of non-crystalline solids by diffraction (Warren *et al.*, 1936 ▸; Warren, 1969 ▸). It has been widely used in the glass and liquid community, where it is often called the radial distribution function (RDF). The term RDF, however, is usually used in describing a single-particle radial distribution, for instance the shell structure in the earth. In order to emphasize the two-body nature of the correlation, the term PDF is more widely used today. Initially this method suffered from a termination error which originates from the limited range of the momentum transfer, *Q*, in the diffraction experiment. But, because of the advent of synchrotron radiation sources and pulsed neutron sources, the energy range of the incident particles has drastically increased. Now *S*(*Q*) can be determined up to *Q* = 35–40 Å^−1^, practically eliminating termination effects. It is now widely used in local structural determination of complex crystals and crystals with disorder, in addition to liquids and glasses (Egami & Billinge, 2012 ▸).

However, the real novelty of this method lies in its capability to bridge different lengthscales, from local through mesoscopic to long-range structures. This capability originates from the inherent high resolution of the technique. The *Q* resolution, *ΔQ*/*Q*, of a powder diffractometer is of the order of 10^−3^ for neutrons and 10^−4^ for synchrotron X-rays, so that the PDF can be determined up to several hundreds of ångstöms as shown in Fig. 1 ▸ (Chung *et al.*, 2005 ▸). In this case (LiNiO_2_ powder), the temperature dependences at short distances (Fig. 1 ▸ *b*) and long distances (Fig. 1 ▸ *c*) are inverted because of nanoscale domain formation. The PDF method was also found to be powerful in determining the particle size and structure of nano-particles (Egami & Billinge, 2012 ▸).

In the article by Marco Scavini *et al.* (Scavini *et al.*, 2015 ▸), the authors very effectively use this method, coupled with electron spin resonance (ESR), to reveal chemical and structural heterogeneity and hierarchical structure in a Ce_1 − *x*_Gd_*x*_O_2 − *x*/2_ solid solution. Replacing Ce^4+^ in CeO_2_ with Gd^3+^ and ½ oxygen vacancy improves the performance of this solid as an electrolyte (Inaba, 1996 ▸). Its crystal structure changes rather smoothly from the fluorite (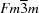) for CeO_2_ to the C-type (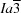) for Gd_2_O_3_ through an intermediate region (C*) in which the superlattice peaks are broader. Upon looking closer in real space using PDF analysis, however, the structure is found to be more complex and heterogeneous. This is not surprising because the Ce^4+^ ion prefers eight-oxygen-coordination, whereas Gd^3+^ prefers six. Therefore a Gd^3+^ ion likes to have another Gd^3+^ ion as a neighbor to share an oxygen vacancy. Thus, the Ce/Gd mixture is not random, but Ce and Gd ions tend to segregate. However, at high temperatures where these structures are formed the entropy effect prevents massive segregation, if the driving force for segregation is not too large. Consequently, they form nanoscale aggregates, the size distribution of which depends on concentration. The phase change from the fluorite to the C-type at *x*_Gd_ = 0.31 can be explained well in terms of percolation of Gd clusters. The energy of a chemical bond is usually of the order of eV, far greater than *k*_B_ *T*. Thus, the environment of each atom tends to be unchanged even in alloys and solid solutions, and compromise for mixing occurs on the mesoscopic lengthscale. This is why a hierarchical structure, such as the one studied by Scanvini *et al.*, is frequently observed in mixed systems. In this case such a hierarchical structure is apparently beneficial to its performance as electrolyte. Hierarchies are not only seen in human societies.

## Figures and Tables

**Figure 1:**
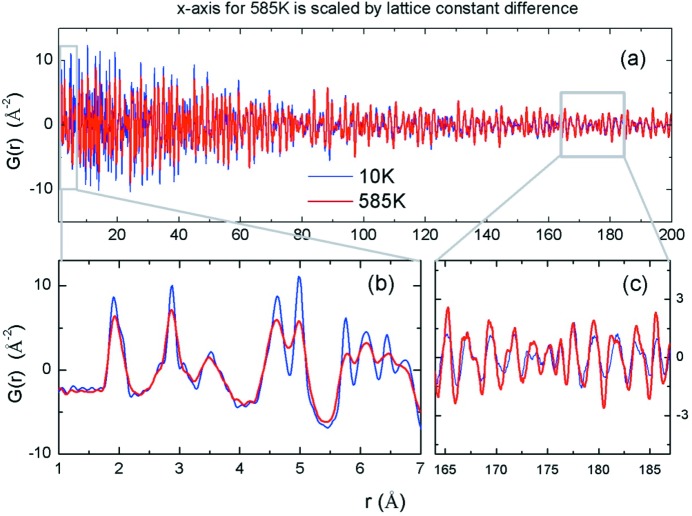
PDF of LiNiO_2_ determined by pulsed neutron diffraction. At short-range the PDF peak height decreases with temperature because of thermal vibration, but at distances greater than 100 Å the trend reverses because of nano-domain formation (Chung *et al.*, 2005 ▸).
